# Reinterpretation of the nomenclatural type of *Pseudobombax heteromorphum* (Malvaceae, Bombacoideae) reveals an overlooked new species from Bolivia

**DOI:** 10.3897/phytokeys.21.5213

**Published:** 2013-05-09

**Authors:** Jefferson G. de Carvalho-Sobrinho, Luciano P. de Queiroz, William S. Alverson

**Affiliations:** 1 Herbário HUEFS, Universidade Estadual de Feira de Santana, Avenida Transnordestina s/n, Novo Horizonte, 44036-900, Feira de Santana, Bahia, Brazil; 2 Department of Botany, Birge Hall, University of Wisconsin-Madison, 53706, Madison, WI, U.S.A.

**Keywords:** *Bombax*, Chiquitano dry forest, new species, seasonally dry neotropical forest, *Tabebuia*, typification

## Abstract

In the course of a taxonomic revision of *Pseudobombax* Dugand, one of us (JGCS) frequently has observed herbarium specimens of Bombacoideae that comprise a mixture of different Angiosperm families. In particular, *Pseudobombax heteromorphum* (Kuntze) A. Robyns, a frequent name in checklists of the Bolivian flora, is based on type material of *Bombax heteromorphum* Kuntze that is clearly a mixture of *Pseudobombax* flowers and *Tabebuia* Gomes ex DC. (Bignoniaceae) leaves. We herein designate as the lectotype of *Bombax heteromorphum* the flowers of an herbarium sheet deposited in NY and as epitype a complete specimen (leaves, flowers, and fruit) in HUEFS. We consider *Bombax heteromorphum* to be a synonym of *Pseudobombax longiflorum* (Mart.) A. Robyns, a species widespread in Neotropical seasonally dry forest of Bolivia, Brazil, Paraguay, and Peru. Furthermore, we describe a new species, *Pseudobombax pulchellum* Carv.-Sobr., apparently endemic to seasonally dry tropical forest (SDTF) in Bolivia (Chiquitano dry forest), based on specimens commonly but incorrectly identified as *Pseudobombax heteromorphum*.We also comment on the morphology, distribution, and conservation status of this new species.

## Abstract (es)

En el curso de una revisión taxonómica de *Pseudobombax* Dugand, uno de nosotros (JGCS) con frecuencia se ha observado especímenes de herbario de Bombacoideae que comprenden una mezcla de diferentes familias de angiospermas. En particular, *Pseudobombax heteromorphum* (Kuntze) A.Robyns, un nombre frecuente en las listas de la flora boliviana, se basa en el material tipo de *Bombax heteromorphum* Kuntze que es claramente una mezcla de flores de *Pseudobombax* y hojas de *Tabebuia* Gomes ex DC. (Bignoniaceae). Aquí se designa como el lectotipo de *Bombax heteromorphum* las flores de uno espécimen de el herbario NY y como epitipo uno espécimen completo (hojas, flores y frutos) de el herbario HUEFS. Consideramos *Bombax heteromorphum* como sinónimo de *Pseudobombax longiflorum* (Mart.) A.Robyns, una especie ampliamente distribuida en los bosques neotropicales estacionalmente seco de Bolivia, Brasil, Paraguay y Perú. Por otra parte, se describe una especie nueva, *Pseudobombax pulchellum* Carv.-Sobr., aparentemente endémica de los Bosques secos Chiquitanos en Bolivia, con base en colecciones comúnmente, pero incorrectamente identificado, como *Pseudobombax heteromorphum*, con comentarios sobre su morfología, distribución y estado de conservación.

## Introduction

**Figure 1.**
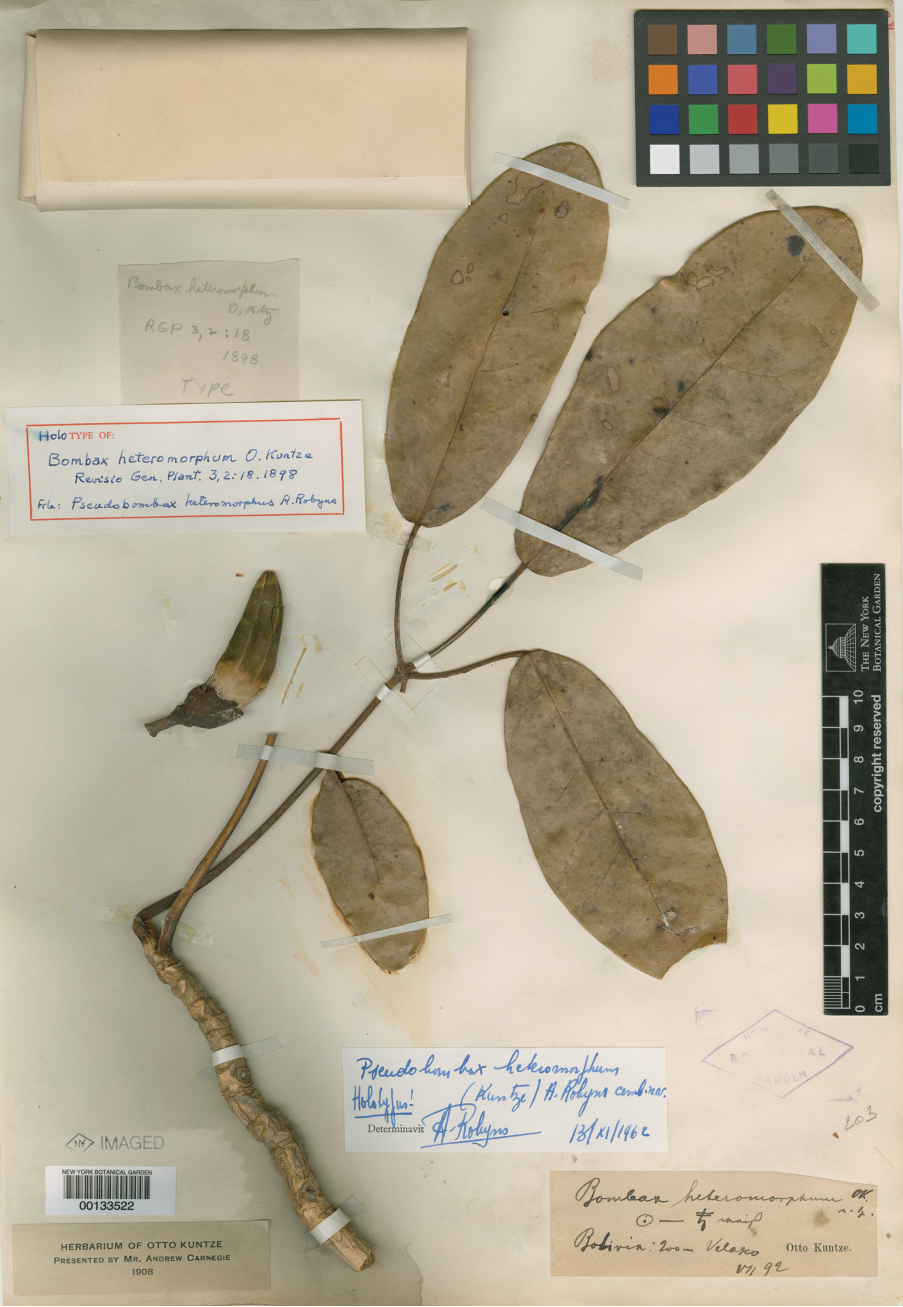
Photograph of the holotype of *Bombax heteromorphum* Kuntze (NY 133522).

In his revision of *Bombax* L. *s.l*., Robyns (1963) transferred *Bombax heteromorphum* Kuntze to *Pseudobombax* Dugand and considered *Pseudobombax heteromorphum* (Kuntze) A.Robyns to be endemic to Bolivia. Based solely on the holotype of *Bombax heteromorphum* (*Kuntze s.n*., Fig. 1), Robyns described *Pseudobombax heteromorphum* as having long petiolules and scarcely expanded petiole apices. In his key to the species of *Pseudobombax*, he used these two characters to separate this speciesfrom morphologically closely related congeners. According to Robyns’s description, *Pseudobombax heteromorphum* also has grooved (canaliculate) petiolules, which if true would represent a unique character in the genus *Pseudobombax*.

In the course of a taxonomic revision of *Pseudobombax* (Carvalho-Sobrinho, in prep.), mixed collections of Bombacoideae, including type specimens based on mixtures, often have been observed. This is especially common for collections from seasonally dry Neotropical forest habitats where species often are leafless during the flowering period. In the present case, a careful examination of the morphology of the leaves and flowers of the holotype of *Bombax heteromorphum* revealed that the specimen is a mixture of reproductive and vegetative elements from two different Angiosperm families. Robyns (1963) failed to note that his type specimen has leaflets that are articulated with the petiole, a character state unknown in *Pseudobombax*; the genus is separated from all other Bombacoideae by the synapomorphy of non-articulated leaflets, which lack an abscission zone between the apex of the petiole and the base of the petiolules (Robyns 1963, Carvalho-Sobrinho and Queiroz 2011).

The holotype of *Bombax heteromorphum* (NY) and an image of an isotype (B as F negative 9535; Field Museum 2012) show floral material matching that of *Pseudobombax longiflorum* (Mart.) A.Robyns, including an androecium with a glabrous, relatively long staminal tube, filaments freely originating from the apex of the tube (i.e., without phalanges), and typical hippocrepiform anthers. However, the vegetative material of these two type specimensmatches the leaves of *Tabebuia aurea* (Silva Manso) Benth. & Hook. f. ex S. Moore (Bignoniaceae), with suberous branches, opposite leaves (inferred from leaf scars), longer and grooved petiolules, leaflets with a clear abscission zone, and a clearly different venation pattern (Fig. 1). *Pseudobombax longiflorum*, a species widespread in Neotropical seasonally dry forest of Bolivia, Brazil, Paraguay, and Peru, and *Tabebuia aurea* are sympatric in the Bolivian Chiquitano dry forest and the latter species has been collected several times in the type locality of *Bombax heteromorphum* (municipality of Velasco, Santa Cruz, Bolivia) according to Tropicos (2012) and specimen labels from herbaria (F, US, and WIS).

## Lectotypification of *Bombax heteromorphum*

Having demonstrated above that the holotype of *Bombax heteromorphum* represents a mixture of leaves belonging to *Tabebuia aurea* and detached flowers belonging to a species of *Pseudobombax* it becomes necessary to select a lectotype from these two elements in order to fix the application of the name (McNeill et al. 2012, Art. 9.14). We choose to lectotypify the name on the flowers only because the name *Bombax heteromorphum* always has been associated with Malvaceae.

***Pseudobombax longiflorum*** (Mart.) A. Robyns, Bull. Jard. Bot. État Bruxelles 33: 57. 1963.

= *Bombax heteromorphum* Kuntze, Revis. Gen. Pl. 3 (3): 18. 1898. *Pseudobombax heteromorphum* (Kuntze) A.Robyns, Bull. Jard. Bot. État Bruxelles 33: 80. 1963. **Lectotype (designated here)**: BOLIVIA. Velasco, *O.Kuntze s.n*. (NY flowers only!). **Epitype (designated here):** BRAZIL. Bahia, Inhaúmas, ca. 10 km de Inhaúmas na estrada para Santa Maria da Vitória, Cerrado, 13°13'47"S, 44°33'09"W, 600 m alt., 15 Aug 2005, *Carvalho-Sobrinho & Queiroz 577* (HUEFS 100549!, lf, fl, fr, in two sheets).

### A new species of *Pseudobombax* long confused with *P. heteromorphum*

Robyns (1963) and subsequent authors (Killeen et al. 1993, Jardim et al. 2003) assigned specimens of a distinctive Bolivian species of *Pseudobombax* to *Pseudobombax heteromorphum* in error. These collections in fact represent a new species, which we describe here.

#### Pseudobombax pulchellum

Carv.–Sobr. sp. nov.

urn:lsid:ipni.org:names:77128380-1

http://species-id.net/wiki/Pseudobombax_pulchellum

Figs 2 , 3

##### Diagnosis.

Similar to *Pseudobombax longiflorum* (Mart.) A.Robyns by its long petiolules, obovate to suborbicular leaflets, and maculate seeds, but differing by the smaller leaves, flowers and fruits, slender branches, petioles 4 times the length of the petiolules (vs. a petiole/petiolule ratio of 6–12 in *Pseudobombax longiflorum*), and fruits acuminate for the distal 20% of their length (vs. 3%–5% in *Pseudobombax longiflorum*).

##### Type.

BOLIVIA. Santa Cruz: 42 km E of Curuyuqui, 18°45'56"S, 62°13'59"W, 350 m, 25 October 1991 (lf, fr), *A. Gentry, R. Foster & M. Peña 75227* (holotype: MO!; isotypes: F!, LPB!, USZ!, WIS!).

##### Description.

Treelets3–8 m, deciduous; branches glabrous, relatively slender, often covered with pale wax; brachyblasts absent. Stipules not seen. Leaves palmately compound, clustered at apex of the branches; petioles (17–) 34–87 mm long, flattened, slender, glabrous, usually glaucous with pale wax at the ends, bases slightly thickened, apices slightly thickened to 3–4 mm diam.; petiolules 14–22 mm long; leaflets (4) 5 (7), chartaceous, proximal leaflets 17–38 × 9–30 mm, distal leaflets 45–57 (–75) × 29–51 mm, obovate, broadly elliptic to suborbicular, apices retuse, rarely acuminate, bases obtuse, rounded, truncate to slightly cordate, margins entire, glabrous on both surfaces, except for sparse, peltate microtrichomes, abaxial surface dull-brown in dried state, midrib prominent abaxially, 8–12 secondary veins inconspicuous, intersecondary veins present, tertiary veins reticulate. Complete inflorescences not seen; pedicels 19 mm long (–26 mm when in fruit). Flowers c. 65 mm long; receptacle with single whorl of c. 5 glands; calyces 8–9 × 11–15 mm, cupular to campanulate, truncate, outwardly glabrous except for peltate microtrichomes, internally sericeous; petals (50–) 61–70 × 6–8 mm, linear to lanceolate, apex acute, dark-brown externally, covered with tufted, rigid hairs, inwardly pilose to glabrescent towards the base, covered mainly by verrucose microtrichomes; stamens c. 150–200, staminal tube 9–10 × 4 mm, pubescent, phalanges absent, filaments free for 40–58 mm, anthers hippocrepiform c. 2 mm long; ovary 5 × 2 mm, oblong-obovoid, glabrous except for peltate microtrichomes, style c. 70 mm long, glabrous, stigma inconspicuously 5-lobed. Capsules c. 90 mm long, woody, oblong-obovoid, conspicuously acuminate for distal 15 mm or so, valves coriaceous, glabrous, kapok abundant, golden brown. Seeds c. 5 mm diam., subglobose to pyriform, maculate, glabrous.

**Figure 2.**
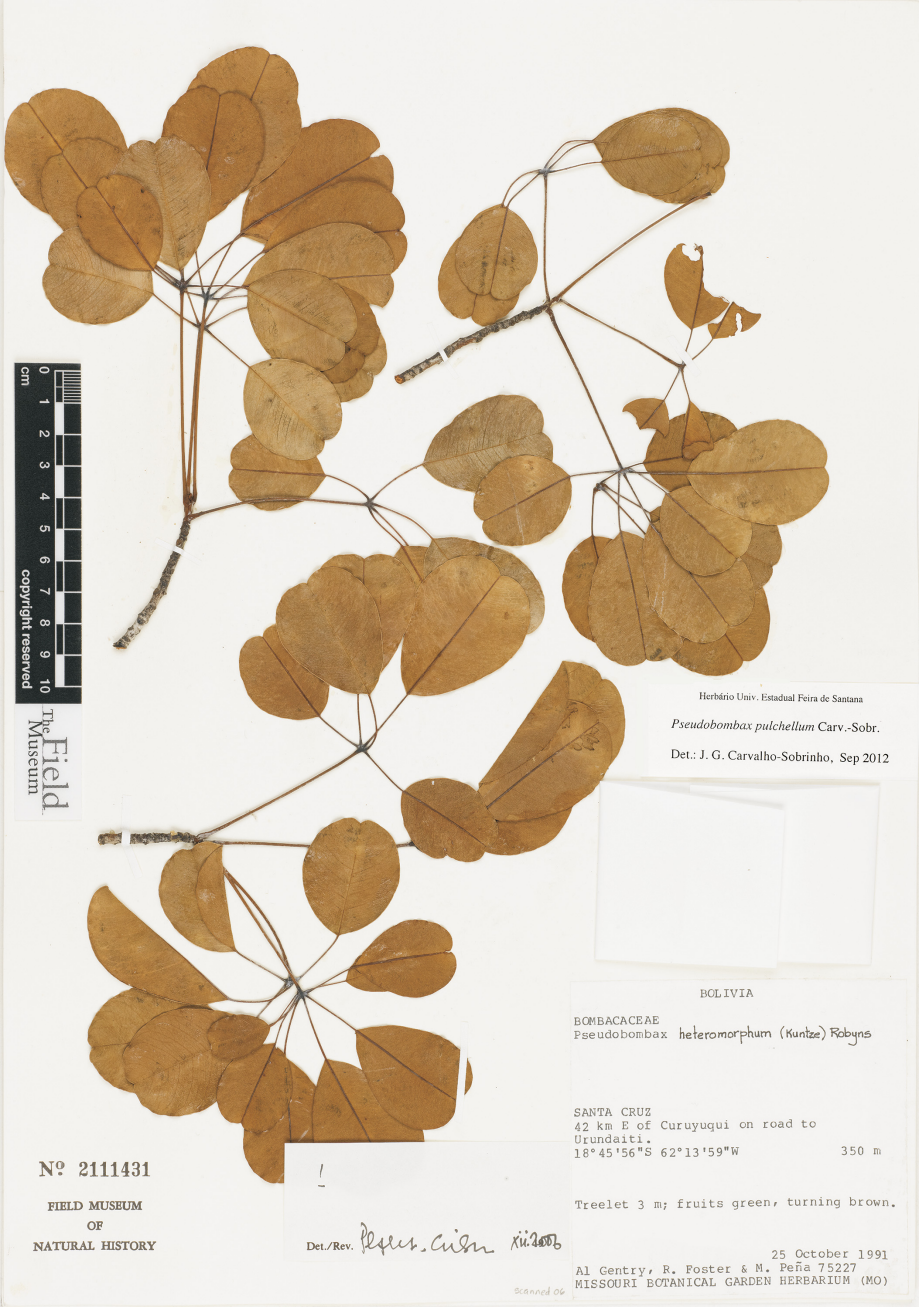
Photograph of an isotype of *Pseudobombax pulchellum* Carv.-Sobr. (*Gentry* et al.75227, F 2111431).

**Figure 3.**
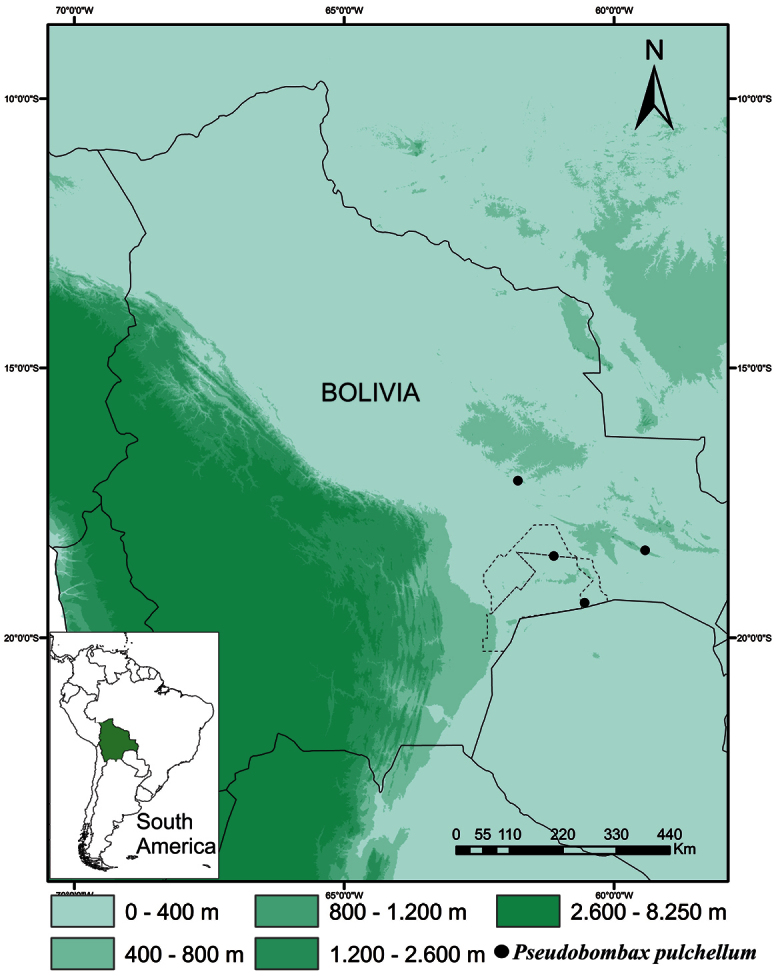
Distribution of *Pseudobombax pulchellum* Carv.-Sobr. in Santa Cruz, Bolivia. Dashed lines indicate the boundaries of the Kaa-Iya del Gran Chaco National Park.

##### Distribution.

*Pseudobombax pulchellum* is known from only four sites in the Department of Santa Cruz, Bolivia, and appears to be endemic to Chiquitano dry forest at elevations of 230 to 520 m.

##### Phenology.

Flowers of this new species are known from a single collection made in June; fruiting material was collected in October.

##### Etymology.

The specific epithet refers to the small, delicate leaflets and long, slender petiolules of this species, diagnostic even in sterile specimens. The epithet also honors the Brazilian botanist Aline Costa da Mota for her important insights and collaboration on the systematics of Bombacoideae.

##### Conservation status.

Although *Pseudobombax pulchellum* occurs in the Kaa-Iya del Gran Chaco National Park, itmust be considered Near Threatened because it is known from only four sites and “there are plausible events that may cause the species to decline, but these are unlikely to make the species Extinct or Critically Endangered in a short time” (IUCN 2010). Extensive fieldwork in other areas of dry forests in Bolivia is necessary to properly survey and to assess the status of this species.

##### Specimens examined.

**BOLIVIA. Santa Cruz**: Chiquitos, 19–23 Dec 1993 (fr), *G. Navarro Sanchez 2192* (LPB!); Cordillera, 09 January 1993 (lf), *G. Navarro Sanchez 1713* (MO!, USZ!); *ibidem*, 18°29'20"S, 61°07'06"W, 230 m, 17 June 1998 (fl), *Alfredo F. Fuentes & G. Navarro Sanchez 2436* (MO!); Ñuflo de Chavez, 17°05'00"S, 61°47'00"W, 400 m, 24 October 1995 (lf), *Alfredo F. Fuentes 1132* (LPB!, USZ,WIS!).

##### Discussion.

*Pseudobombax pulchellum* is a remarkable species by its diminutive aspect, especially the relatively small, retuse, obovate to suborbicular leaflets, and the flowers; the petiolules are markedly long in relation to the petiole, and fruits are conspicuously acuminate. It seems to be closely related to *Pseudobombax longiflorum* (Mart.) A. Robyns, a sympatric congener in Bolivian Chiquitano dry forest. The two are similar because of their glabrous aspect, long petiolules, leaflets with retuse apices, truncate to cordate bases, and maculate seeds.

The new species is also similar to *Pseudobombax croizatii* A.Robyns and *Pseudobombax minimum* Carv.-Sobr. & L.P. Queiroz; both of these species share small flowers (60–65 mm long), glabrous staminal tubes, androecia with relatively few stamens (c. 150–200), and glabrous fruits. The following key can be used to separate these four species of *Pseudobombax*.

**Table:** 

1	Petiolules 14–75 mm long. Petals externally blackish, staminal tube lacking phalanges (filaments freely originating from the apex of the tube). Seeds bicolored, maculate	2
–	Petiolules to 5 mm long. Petals externally cream-colored, staminal tube originating phalanges 3–5 mm long. Seeds uniformly colored	3
2	Petioles 4 times the length of the petiolules. Flowers c. 65 mm long, calyx 8–9 mm long, staminal tube c. 10 mm long. Capsules c. 90 mm long, acuminate for the distal 20% of their length	*Pseudobombax pulchellum* (Bolivia)
–	Petioles 6–12 times the length of the petiolules. Flowers 15–22 mm long, calyx 15–25 mm long, staminal tube 35–60 mm long. Capsules 14–24 mm long, acuminate for the distal 3%–5% of their length	*Pseudobombax longiflorum* (Bolivia, Brazil, Paraguay, Peru)
3	Leaflets 7–9, obovate, cuneate, margins revolute. Flowers to 60 mm long, staminal tube glabrous. Capsules 55 mm long, not acuminate	*Pseudobombax minimum* (Central Brazil)
–	Leaflets 5, elliptic to broad-elliptic, acute, margins plane. Flowers 85–100 mm long, staminal tube with bands of simple trichomes. Capsules c. 70 mm long, acuminate	*Pseudobombax croizatii* (Colombia, Venezuela)

## Supplementary Material

XML Treatment for Pseudobombax pulchellum
